# Fusion-Extracted Features by Deep Networks for Improved COVID-19 Classification with Chest X-ray Radiography

**DOI:** 10.3390/healthcare11101367

**Published:** 2023-05-10

**Authors:** Kuo-Hsuan Lin, Nan-Han Lu, Takahide Okamoto, Yung-Hui Huang, Kuo-Ying Liu, Akari Matsushima, Che-Cheng Chang, Tai-Been Chen

**Affiliations:** 1 Department of Information Engineering, I-Shou University, Kaohsiung City 82445, Taiwan; ed104973@edah.org.tw; 2 Department of Emergency Medicine, E-DA Hospital, I-Shou University, Kaohsiung City 82445, Taiwan; 3 Department of Pharmacy, Tajen University, Pingtung City 90741, Taiwan; 4 Department of Radiology, E-DA Cancer Hospital, I-Shou University, No. 1, Yida Road, Jiao-su Village, Yan-Chao District, Kaohsiung City 82445, Taiwan; ed102500@edah.org.tw; 5 Department of Medical Imaging and Radiological Science, I-Shou University, Kaohsiung City 82445, Taiwan; yhhuang@isu.edu.tw; 6 Department of Radiological Technology, Faculty of Medical Technology, Teikyo University, Tokyo 173-8605, Japan; okamoto@med.teikyo-u.ac.jp (T.O.); matsushima_a@med.teikyo-u.ac.jp (A.M.); 7 Department of Radiology, E-DA Hospital, I-Shou University, Kaohsiung City 82445, Taiwan; ed104721@edah.org.tw; 8 Institute of Statistics, National Yang Ming Chiao Tung University, Hsinchu 30010, Taiwan

**Keywords:** CXR, COVID-19, bacterial pneumonia, hybrid AI model, deep learning, feature fusion, support vector machine

## Abstract

Convolutional neural networks (CNNs) have shown promise in accurately diagnosing coronavirus disease 2019 (COVID-19) and bacterial pneumonia using chest X-ray images. However, determining the optimal feature extraction approach is challenging. This study investigates the use of fusion-extracted features by deep networks to improve the accuracy of COVID-19 and bacterial pneumonia classification with chest X-ray radiography. A Fusion CNN method was developed using five different deep learning models after transferred learning to extract image features (Fusion CNN). The combined features were used to build a support vector machine (SVM) classifier with a RBF kernel. The performance of the model was evaluated using accuracy, Kappa values, recall rate, and precision scores. The Fusion CNN model achieved an accuracy and Kappa value of 0.994 and 0.991, with precision scores for normal, COVID-19, and bacterial groups of 0.991, 0.998, and 0.994, respectively. The results indicate that the Fusion CNN models with the SVM classifier provided reliable and accurate classification performance, with Kappa values no less than 0.990. Using a Fusion CNN approach could be a possible solution to enhance accuracy further. Therefore, the study demonstrates the potential of deep learning and fusion-extracted features for accurate COVID-19 and bacterial pneumonia classification with chest X-ray radiography.

## 1. Introduction

The coronavirus disease 2019 (COVID-19) infection has affected millions of people worldwide, causing significant economic, social, and health consequences. Early and accurate disease detection is essential to prevent its spread and provide timely treatment. Chest X-rays are one of the diagnostic tools used to detect COVID-19 infection. The difficulty in distinguishing between COVID-19 and bacterial phenomena in chest X-ray radiography can be challenging. COVID-19 and bacterial pneumonia can present similar imaging features on chest X-rays, such as consolidations, ground-glass opacities, and reticular patterns [1,2]. These overlapping features can make it difficult for radiologists to accurately differentiate between the two conditions based on chest X-rays alone. Some patients may simultaneously have COVID-19 and bacterial pneumonia, which can further complicate the interpretation of chest X-rays [3]. In such cases, it may be challenging to determine the predominant cause of the patient’s respiratory symptoms based on imaging findings alone. The appearance of COVID-19 and bacterial pneumonia on chest X-rays can change as the disease progresses. A patient’s chest X-ray might initially show findings suggestive of one condition but later develop features more characteristic of the other. This variability can make establishing a definitive diagnosis based on a single X-ray challenging. The quality of chest X-ray images can be affected by factors such as patient positioning, exposure settings, and motion artifacts [4]. Poor image quality can hinder the radiologist’s ability to identify subtle imaging findings that might help differentiate between COVID-19 and bacterial pneumonia. Accurately interpreting chest X-ray findings in COVID-19 and bacterial pneumonia relies heavily on the experience and expertise of the radiologist. Inexperienced radiologists may have difficulty distinguishing between the two conditions, especially in cases with subtle or atypical imaging findings. Chest X-rays provide a limited view of the lungs and are less sensitive than other imaging modalities, such as computed tomography (CT) scans, in detecting specific lung abnormalities. This limitation can make it difficult to differentiate between COVID-19 and bacterial pneumonia based solely on chest X-ray findings [5,6,7,8,9,10]. The studies focused on improving the detection of COVID-19 using chest X-ray images, aiming to minimize false results and support medical professionals in early diagnosis [11,12].

Therefore, developing an automated COVID-19 classification system using chest X-rays can aid in the rapid and accurate diagnosis of the disease. Deep learning has shown significant promise in various medical image analysis tasks, including detecting COVID-19 from chest X-ray radiography [13]. Convolutional neural networks (CNNs) have been widely used to extract features from medical images, and their performance can be further enhanced by using transferred learning. Transferred learning involves taking a pre-trained network on a large dataset and fine-tuning it for a specific task, such as COVID-19 classification [14,15,16,17,18,19].

Various machine learning techniques have been proposed to overcome the difficulty in distinguishing between COVID-19 and bacterial phenomena [4,5,6,20]. These techniques involve using deep learning algorithms to extract features from chest X-ray images and classify them. For example, in the study mentioned in the introduction, a hybrid artificial intelligence (AI) model was developed using multiple CNN models and a SVM classifier to classify chest X-ray images into normal, COVID-19, and bacterial categories.

Hence, the references highlight the potential of deep learning for COVID-19 classification using chest X-ray images. Various deep learning architectures have been used to achieve high accuracy in COVID-19 classification. In recent years, numerous studies have explored using deep learning for COVID-19 classification of chest X-rays. Transferred learning and fusion of features have been shown to improve classification performance. However, most of these studies focused on using a single deep learning architect.

This paper is organized as follows: Section 1 provides an introduction to the study, outlining the motivation and objectives. Section 2 reviews the related works on the classification of COVID-19 using deep learning methods, including both single deep learning methods and fusion-based models. Section 3 describes the materials and methods used in the study, including the research flow, datasets, image processing techniques, the application of transferred learning for convolutional neural networks, and the performance index for classification. Section 4 presents the results of the study, followed by a discussion in Section 5. Finally, Section 6 concludes the paper, summarizing the key findings and contributions of the study.

## 2. The Related Works of Classification COVID-19 Using Deep Learning Methods

As 2020 commenced, numerous studies emerged that focused on employing AI-based (artificial intelligence) models and radiological images for the detection of the novel coronavirus. These methods can be broadly categorized into two distinct groups. The first group comprises approaches based on single deep learning methods, while the second group encompasses fusion-based methods. In this section, we will explore the related works within these two classifications.

### 2.1. Single Deep Learning Method

In various studies, individual CNNs have been utilized for different purposes. For instance, CoroNet was employed to detect COVID-19 through X-ray images, achieving an 89.6% accuracy rate. The promising outcomes suggest that this model could significantly assist doctors worldwide in combating the COVID-19 pandemic [4]. In another study [21], researchers combined numerous pre-trained CNN methods to construct cascaded deep learning classifiers, obtaining an accuracy of 99.9% in identifying COVID-19 cases. In a study [22], researchers diagnosed COVID-19 by applying five pre-trained CNN methods with transfer learning techniques and achieved a 96.0% accuracy rate. In a study [23], four different CNN methods were independently employed, incorporating data augmentation techniques to diagnose COVID-19. Among them, VGG19 achieved the highest accuracy of 90.5%.

Therefore, when applied to create a CNN model, the transfer learning technique is a widely used approach for developing CNN-based image classifiers.

### 2.2. Fusion-Based Models

Fusion-based CNN methods have been extensively used to support COVID-19 diagnosis. In a study [24], four radiomics techniques were applied to analyze the original CT and X-ray images. Three separate CNN models were trained on distinct sets of radiomics, X-ray, and CT images. Deep features were extracted from the CNN models, and their dimensions were reduced using the Fast Walsh Hadamard Transform, leading to a spectral-temporal representation of COVID-19 patterns. Ultimately, four classifiers were built using the combined features, achieving an accuracy of 99.0%. In other studies [25,26,27], spatial information was extracted using CNN methods, while spectral–temporal information was obtained by applying transform methods. A machine learning model was built based on these extracted features, resulting in high accuracy for classification achieved by these methods.

In a study [28], the authors presented a novel fusion model that combines hand-crafted and deep learning features, called the FM-HCF-DLF model, for diagnosing and classifying COVID-19. The proposed FM-HCF-DLF model consisted of three main processes: Gaussian filtering-based preprocessing, feature extraction through fusion modeling, and classification. The accuracy of the presented model was 94.08%. A study [29] extracted the predictive features from original images using CNN and co-matrix images using algebraic methods. In this case, the presented model’s accuracy was 99.0%.

As we approach the era of lifting COVID-19 restrictions, routine COVID-19 screening may no longer be necessary. However, the treatment for severe pneumonia caused by COVID-19 significantly differs from that of bacterial pneumonia. Consequently, the ability to accurately diagnose COVID-19 pneumonia using high-precision chest X-ray imaging will hold great clinical value in the future.

## 3. Materials and Methods

### 3.1. The Flow of Research

**Figure 1:**
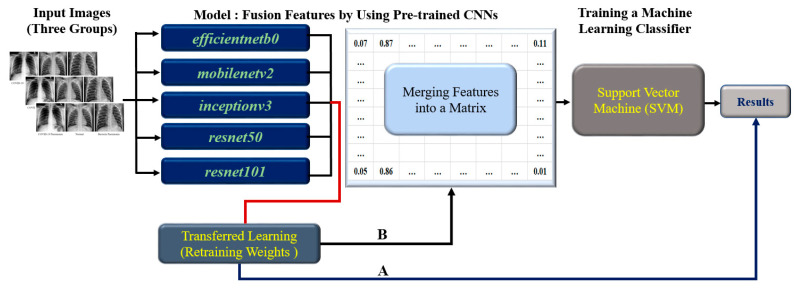
The flowchart demonstrates the process followed in this study. A represents the convolutional neural networks (CNN) with transferred learning, and B shows the fusion of extracted features combined with a support vector machine (SVM) classifier.

The flow of this research is illustrated in Figure 1 and includes the following steps: case collection, image preprocessing, model creation, training the support vector machine (SVM) classifier, and evaluation of results. The CNN models used in the study consist of EfficientNetB0, MobileNetV2, InceptionV3, ResNet50, and ResNet101. The design of the classification model involved two main steps: (A) transferred learning, which adapts a pre-trained CNN model to the specific problem at hand, and (B) feature extraction, where the model extracts relevant features from the data. These extracted features are then merged into a feature matrix to train an SVM classifier, which performs the final classification task.

The flowchart demonstrates the process followed in this study. The steps to train and test the classifier can be summarized as follows:

1. Image preprocessing: Prepare chest X-ray images for input, including image resizing and normalization.
2. Transferred Learning: Train each of the five individual CNN models (EfficientNetb0, MobileNetv2, Inceptionv3, ResNet50, and ResNet101) using transferred learning on the preprocess chest X-ray images.
3. Feature Extraction: Extract features from each CNN model’s output layer.
4. Feature Fusion for training model using 80% of the dataset: A. Split the dataset into training (80%) and testing (20%) sets. B. Combine the features extracted from each CNN model using the training set. C. Create a feature matrix with dimensions 15 × 4208 for the training set, which combines all five individual CNN model features (each contributing a 3 × 4208 feature size). D. Proceed to train the SVM classifier using the fused feature matrix from the training set.
5. Training and testing SVM Classifier: Use the fused feature matrix as input for training the SVM classifier with an RBF kernel. The fused feature matrix size is 15 × 1052 for testing the SVM classifier.
6. Evaluated Classifier Performance: A. Apply the trained SVM classifier on the test dataset to predict the class labels (COVID-19, Normal, or Bacterial). B. Calculate performance metrics such as accuracy, Kappa values, recall rate, precision scores, and ROC area.

### 3.2. The Datasets

**Figure 2:**
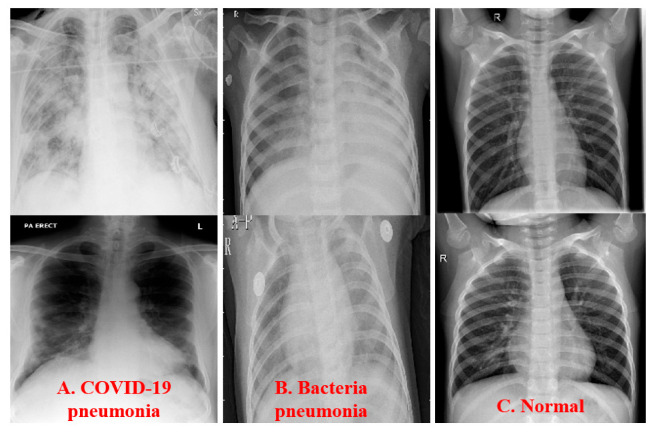
Example images from the three groups ((**A**.) COVID-19, (**B**.) Bacterial Pneumonia, and (**C**.) Normal).

The dataset used in this study collects open public images to investigate the Fusion CNN model (URL: https://www.kaggle.com/datasets/paultimothymooney/chest-xray-pneumonia, accessed on 13 June 2022) [30]. A total of 5260 chest X-ray (CXR) images were successfully collected. The sample size includes 1792 normal images, 1658 COVID-19 images, and 1800 bacterial pneumonia images (Figure 2). In the normal chest X-ray (left panel), the lungs appear clear without abnormal opacities. Bacterial pneumonia (middle) generally displays a localized lobar consolidation, as seen in this example in the right upper lobe (indicated by white arrows). In contrast, viral pneumonia (right) demonstrates a more widespread “interstitial” pattern throughout both lungs.

### 3.3. Image Processing

Two experimental processing of images were applied in this study:

1. The first step is to load the image into memory and convert it to an appropriate format for analysis. This can include resizing the image to a 300 × 300 matrix size and normalizing the pixel values to (0, 1).
2. Gray chest X-ray images can sometimes lack contrast or sharpness, making it difficult for a CNN to identify features. The input chest X-ray images were transformed to RGB three channels (or pseudo-color). This step was performed to enhance the contrast and sharpness of the images, enabling the CNN models to identify relevant features better.

The purpose of image processing is to improve the accuracy of classification via deep learning.

### 3.4. Transferred Learning for Convolutional Neural Network

All used CNN methods are powerful tools for image classification tasks, and the choice of architecture depends on the distinctive needs of the application in terms of accuracy, speed, and computational resources available.

EfficientNetB0 is a family of CNN models known for their high accuracy and low computational cost. They use a compound scaling method that scales the width, depth, and resolution of the neural network in a balanced way. EfficientNetB0 has achieved state-of-the-art performance on a variety of image classification benchmarks.

MobileNetV2 is another efficient CNN architecture designed for mobile and embedded applications. It uses depthwise separable convolutions to reduce the required parameters and computations. MobileNetV2 is fast and accurate and has been used in many applications, including object detection and semantic segmentation.

InceptionV3 is a CNN architecture that combines 1 × 1, 3 × 3, and 5 × 5 convolutions in parallel to capture features at different scales. It also uses a technique called “factorization” to reduce the number of parameters in the network. InceptionV3 is accurate and efficient and has been used in various image classification tasks.

ResNet50 and ResNet101 are part of the ResNet family of CNN models. ResNet50 has 50 layers, while ResNet101 has 101 layers. ResNet models use a “residual connections” technique to address the vanishing gradient problem that can occur in deep neural networks. ResNet models perform well on various image classification benchmarks and are widely used in computer vision applications.

The choice of hyperparameters, such as the number of epochs, batch size, and learning rate, can significantly impact the performance of a machine learning model. The specific values chosen for these hyperparameters in a study are often based on empirical results, prior research, and practical considerations. In this study, the hyperparameter values were defined as below.

1. Thirty epochs: The number of epochs determines how often the entire dataset passes through the training process. The choice of 30 epochs might have been based on previous experiments or research, indicating that this number of epochs provides a good balance between training time and model performance (i.e., avoiding underfitting or overfitting).
2. Batch size of five: The batch size is the number of samples used for each weight update during training. A smaller batch size, such as five, can result in faster convergence of the model and potentially better generalization to new data, as it introduces some noise during training. However, it might require more computation time compared to larger batch sizes. The choice of a batch size of five could be based on prior experience, computational constraints, or the specific dataset used in this study.
3. Learning rate of 0.001: The learning rate determines the step size taken during optimization. A learning rate of 0.001 is common in many deep learning applications, as it often balances convergence speed and stability. This learning rate might have been chosen based on prior research or empirical results, suggesting that it works well for this study’s specific problem and model architecture.

These hyperparameter values might not be optimal for all datasets or model architectures. They may have been chosen based on the specific study context, and other values could yield better results. Hyperparameter tunings, such as grid search or Bayesian optimization, can systematically explore different hyperparameter combinations to find the best set for a given problem.

We used an 80–20 split for our dataset, with 80% of the data (4208 images) used for training the SVM classifier and 20% (1052 images) used for testing. The randomly splitting schema was chosen based on the standard practice in the field to ensure sufficient data for training while maintaining an adequate amount for evaluation.

### 3.5. Performance Index for Classification

The performance of CNN models is assessed using recall rate, precision, accuracy, F1-score, and Kappa values (Cohen’s Kappa). These metrics are used for evaluating the classification performance of CNNs (convolutional neural networks) and other machine learning models because they provide different perspectives on the model’s performance, allowing for a comprehensive understanding of its strengths and weaknesses. In these calculations, TP represents True Positives, FP represents False Positives, TN represents True Negatives, and FN represents False Negatives.

The recall rate (sensitivity or true positive rate) measures the ratio of actual positive cases that the model correctly identifies. It is especially important in situations where false negatives are more critical, such as medical diagnoses, where failing to identify a condition could lead to severe consequences (Equation (1)).

Recall rate = TP/(TP + FN)(1)

Precision (positive predictive value) measures the proportion of positive cases identified by the model that are positive. It is essential when the cost of false positives is high, such as in spam detection, where incorrectly flagging legitimate messages can be detrimental (Equation (2)).

Precision = TP/(TP + FP)(2)

Accuracy measures the proportion of correct predictions (both positive and negative) made by the model out of all predictions. It provides a general overview of the model’s performance. However, accuracy can be misleading in imbalanced datasets, where a model can achieve high accuracy by simply predicting the majority class (Equation (3)).

Accuracy = (TP + TN)/(TP + TN + FP + FN)(3)

Kappa (Cohen’s Kappa) measures the statistical agreement between the model’s predictions and the actual labels while accounting for agreement by chance. It is a more robust metric than accuracy, as it is less affected by class imbalance. A higher Kappa value indicates better classification performance, with 1 representing perfect agreement and 0 representing agreement by chance (Equation (4)).

Kappa = (Observed Accuracy − Expected Accuracy)/(1 − Expected Accuracy)(4)

where Observed Accuracy is the proportion of correctly classified instances (same as accuracy), and Expected Accuracy is the accuracy that would be expected if the predictions were made by chance.

By evaluating a CNN model using these metrics, researchers and practitioners can gain a deeper understanding of the model’s performance, which helps in model selection, hyperparameter tuning, and making informed decisions about deploying the model in real-world applications.

## 4. Results

Table 1 shows the performance of various convolutional neural networks (CNNs) for classifying images into COVID-19, Normal, and Bacterial classes after applying transferred learning. The evaluation metrics used are recall rate, precision, accuracy, and Kappa. Based on the table, Fusion CNN performs the best across all the evaluation metrics. It has the highest recall rate, precision, accuracy, and Kappa score. The results suggest that Fusion CNN is the most suitable model for this classification task among the tested CNNs. When comparing the accuracy and Kappa scores, the Fusion CNN model achieves the highest, with an accuracy of 0.994 and a Kappa score of 0.991. This indicates that the Fusion CNN model is the best-performing model among the evaluated models for this classification task. The second-best performing model is EfficientNetb0, with an accuracy of 0.988 and a Kappa score of 0.983. The other models have lower accuracy and Kappa scores, with ResNet50 being the least performing model with an accuracy of 0.920 and a Kappa score of 0.880.

In Table 2, the confusion matrixes demonstrate the performance of the five transferred learning CNN and Fusion CNN models in classifying images into COVID-19, Normal, and Bacterial classes on merging datasets. The confusion matrix for the Fusion CNN model demonstrates a high classification accuracy, with the majority of images correctly classified (diagonal cells) and a low number of misclassifications (off-diagonal cells). The Fusion CNN model effectively classifies images into COVID-19, Normal, and Bacterial classes.

The Fusion CNN model performs excellently in classifying COVID-19, Normal, and Bacterial pneumonia cases using chest X-ray radiography, with high recall rates, low false positive rates, high precision, and high ROC area values for each group (Table 3). All ROC areas are higher than 0.99, representing an excellent classifier performance, with a value close to one indicating high accuracy.

## 5. Discussion

The Fusion CNN outperforms the other models because it combines the strengths and learned features from the other five CNNs after transferred learning. By merging the features from different models, Fusion CNN can leverage each model’s complementary information, diverse perspectives, and unique strengths to enhance its overall classification performance. Here are some reasons why Fusion CNN performs better than individual models:

1. Complementary information: Different CNN architectures have varied strengths in recognizing specific features or patterns in the data. By merging the features extracted by multiple CNNs, Fusion CNN can access a richer and more comprehensive set of features, which aids in achieving better classification results.
2. Ensemble effect: Combining the outputs of multiple models can help reduce the risk of overfitting the training data and improve the generalization to unseen data. Fusion CNN effectively works as an ensemble method, where the combined predictions of several models lead to a more accurate and robust final prediction.
3. Error correction: If a single CNN model makes a mistake in classification, the other models’ correct predictions can compensate for the error when the features are merged in Fusion CNN. This error correction mechanism can lead to improved performance.

Hence, the Fusion CNN model benefits from the complementary information, ensemble effect, diversity, and error correction provided by combining features from the other five CNNs after transferred learning. This results in superior performance compared to the individual models. The merits include improved accuracy, robustness, efficient use of resources, and transferability. The classification model can be more robust to input data variations by fusing multiple features. For example, the model can handle variations in the position or orientation of the image. In some cases, extracting features from multiple CNN models and fusing them may be more efficient than training a single, more complex model. This can be particularly useful in resource-constrained settings. Fused features may be more easily transferable to other datasets or tasks as they capture more general information about the image rather than task-specific information.

However, there are some pitfalls to using fusion features, including overfitting, complexity, limited interpretability, and the need for large amounts of data. The problem of overfitting would damage the flexibility of trained models. In addition, overfitting is risky when fusing features from multiple models. This can happen if the models are too similar or if the features are not sufficiently independent. Moreover, fusing features could increase the complexity of the model, both in terms of implementation and computation. In order to effectively fuse features from multiple models, large amounts of data may be required to ensure that the models are trained on diverse samples of the population. Therefore, using fusion features in deep learning can be a powerful approach to improving the performance of image classification tasks. However, it is crucial to be aware of the potential pitfalls and to carefully evaluate the trade-offs between complexity, accuracy, and interpretability.

The merits of using fusion features in deep learning models for COVID-19 classification using chest X-ray images include improved classification performance compared to single networks, increased accuracy and robustness to noise and artifacts in the images, improved sensitivity and specificity, and improved robustness to variations in image quality and size. It is important to carefully consider the benefits and limitations of using fusion features in deep learning models for COVID-19 classification. Further research is needed to evaluate different fusion methods’ effectiveness and improve generalizability and computational efficiency.

Table 4 presents information about the Fusion CNN model and the five individual CNN models used for feature extraction via transferred learning. The table lists the models, their types, and the feature size they contribute to the Fusion CNN model. For Fusion CNN, the type is classifier base, which means that it serves as the base classifier for the hybrid AI model. The feature size is 15 × 4208, indicating that the Fusion CNN model takes the combined features from the five CNN models, with each model contributing a 3×4208 feature size, resulting in a total feature size of 15 × 4208.

**Figure 3:**
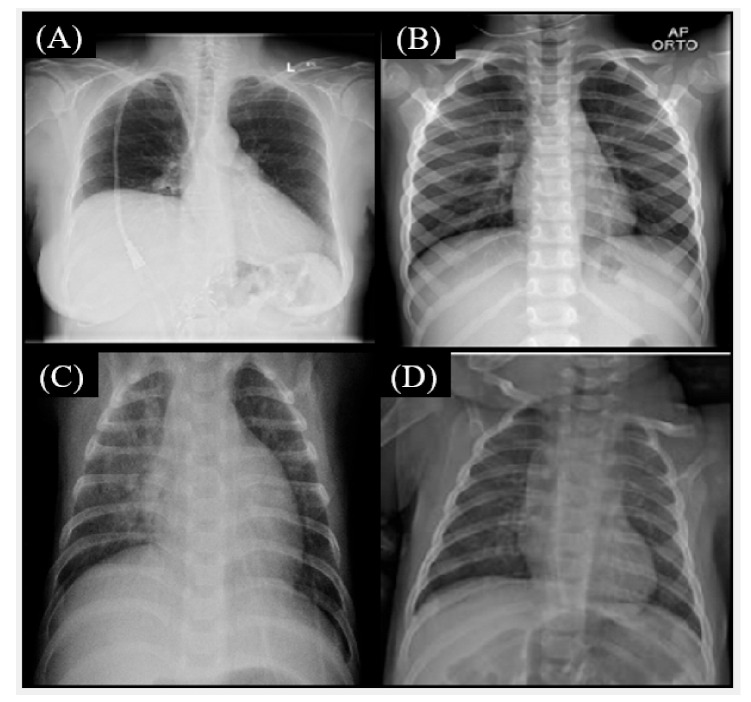
The true label is COVID-19 but Fusion convolutional neural networks (CNN) model was predicted as Normal (**A**,**B**) and Bacterial pneumonia (**C**,**D**).

**Figure 4:**
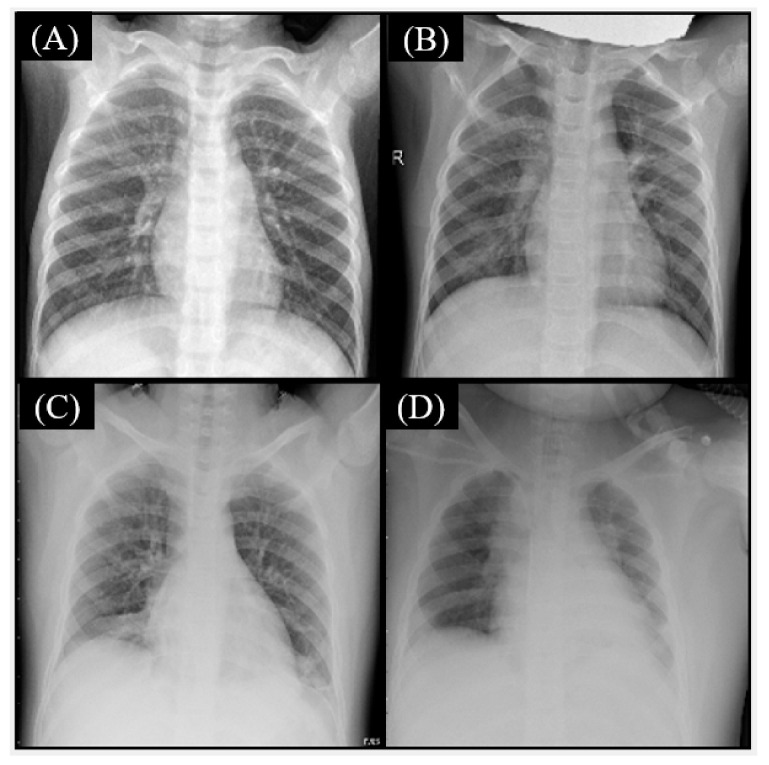
The true label is Bacterial pneumonia, but the Fusion convolutional neural networks (CNN) model was predicted as Normal (**A**,**B**) and COVID-19 (**C**,**D**).

**Figure 5:**
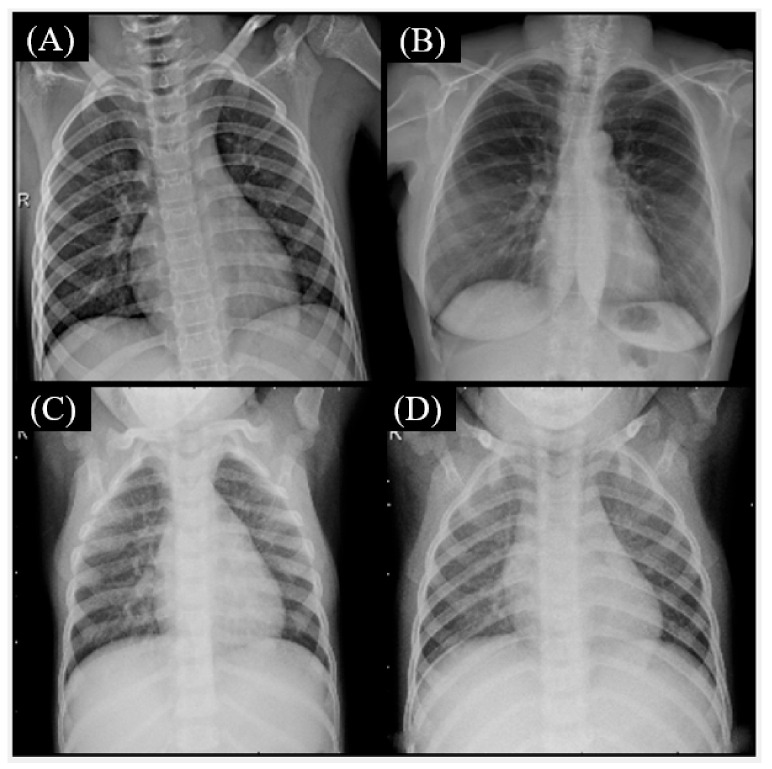
The true label is Normal, but the Fusion convolutional neural networks (CNN) model was predicted as COVID-19 (**A**,**B**) and Bacterial pneumonia (**C**,**D**).

The misclassification using the Fusion CNN model showed a few cases in Figure 3, Figure 4 and Figure 5. The true label is COVID-19 but Fusion CNN model was predicted as Normal (Figure 3A,B) and Bacterial pneumonia (Figure 3C,D). The possible reasons include high similarity with normal chest X-ray images for Figure 3A,B. The textural and brightness patterns are close to bacterial pneumonia for Figure 3C,D.

The true label is bacterial pneumonia, but the Fusion CNN model was predicted as Normal (Figure 4A,B) and COVID-19 (Figure 4C,D). The possible reasons include no obvious patterns shown in chest X-ray images for Figure 4A,B, such as consolidation, air bronchograms, pleural effusion, cavitation, and infiltration. The textural patterns are shown as consolidation, linear opacities, and pleural effusion that have high similarity to COVID-19 for Figure 4C,D.

The true label is Normal chest X-ray, but the Fusion CNN model was predicted as COVID-19 (Figure 5A,B) and Bacterial pneumonia (Figure 5C,D). The image patterns were shown as linear opacities and ground-glass opacities in chest X-ray images for Figure 5A,B. The textural patterns are shown as air bronchograms and cavitation that have high similarity to COVID-19 for Figure 5C,D.

Differential diagnosis between bacterial pneumonia and COVID-19 pneumonia using chest X-ray can be challenging due to several factors.

**Overlapping findings**: Both bacterial and COVID-19 pneumonia can exhibit similar radiographic features, such as consolidation and ground-glass opacities. These overlapping findings can make it difficult to differentiate between the two conditions based on chest X-ray alone.

**Nonspecific findings**: The radiographic findings in both bacterial and COVID-19 pneumonia can be nonspecific and may resemble other lung conditions, such as viral pneumonia, atelectasis, or pulmonary edema. This lack of specificity further complicates the differential diagnosis.

**Variability in the presentation**: The appearance of both bacterial and COVID-19 pneumonia on chest X-ray can vary significantly depending on factors such as the causative organism, the stage of the disease, and the patient’s immune response. This variability adds to the difficulty of distinguishing between the two conditions.

**Limited sensitivity**: Chest X-rays have limited sensitivity in detecting early or mild cases of pneumonia, particularly for COVID-19, where ground-glass opacities can be subtle and easily missed. Consequently, relying solely on chest X-rays for differential diagnosis may lead to false-negative results.

**Image quality and interpretation**: The quality of chest X-ray images can be affected by factors such as patient positioning, exposure, and technique. Moreover, the interpretation of chest X-ray findings can be subjective and may vary between radiologists, leading to inconsistencies in the differential diagnosis.

Due to these challenges, it is essential to consider a comprehensive clinical assessment and additional diagnostic tests such as sputum culture, blood tests, RT-PCR for COVID-19, or a chest CT scan to differentiate between bacterial and COVID-19 pneumonia more accurately.

Meanwhile, the results of the presented method were compared with related works (Table 5). Based on the table, the presented method has the highest reported accuracy of 0.994 among the methods listed, tied with Attallah’s RADIC model. However, it is important to note that the other methods in the table also achieved high levels of accuracy, ranging from 0.896 to 0.999. While some methods may have limitations, such as limited dataset size or computational complexity, they are still valuable contributions to the field of COVID-19 diagnosis.

## 6. Conclusions

This paper highlights the use of chest X-ray images for COVID-19 image classification instead of CT images and demonstrates highly accurate results in detecting the disease. The key novelties of our study include (1). The development and application of the Fusion CNN method combined extracted features from multiple deep learning models to improve accuracy (2). Using a support vector machine (SVM) classifier to further enhance the performance of the Fusion CNN model (3). Demonstrating the potential of chest X-ray images as a viable alternative to CT images for COVID-19 diagnosis.

By leveraging advanced machine learning techniques, the study showcases the potential of chest X-ray images as a reliable alternative to CT images for COVID-19 diagnosis. The results indicate that the fusion CNN models with the SVM classifier provided reliable and accurate classification performance, with Kappa values no less than 0.990. A Fusion CNN approach could be a possible solution to enhance accuracy further. The transferred learning CNNs would be a good model for classifying chest X-rays. The Fusion CNN model with a machine learning classifier provided reliable and accurate performance for classifying CXRs into Normal, Bacterial, and COVID-19 pneumonia categories. A larger CXR dataset should be considered for future clinical use to validate the model’s performance further.

## Figures and Tables

**Table 1:** The performance of classification using convolutional neural networks (CNNs) after transferred learning.

CNN	Recall Rate			Precision			Accuracy	Kappa
	COVID-19	Normal	Bacterial	COVID-19	Normal	Bacterial		
EfficientNetb0	0.995	0.983	0.988	0.990	0.988	0.983	0.988	0.983
MobileNetv2	0.987	0.919	0.964	0.980	0.975	0.918	0.956	0.934
Inceptionv3	0.984	0.964	0.973	0.990	0.970	0.961	0.973	0.960
ResNet50	0.881	0.905	0.972	0.980	0.978	0.832	0.920	0.880
ResNet101	0.984	0.966	0.976	0.988	0.978	0.959	0.975	0.962
Fusion CNN	0.997	0.994	0.992	0.998	0.991	0.994	0.994	0.991

CNN: Convolutional neural networks.

**Table 2:** The confusion matrixes demonstrate the performance of the five transferred learning CNN and one Fusion CNN model in classifying images into COVID-19, Normal, and Bacterial classes.

	Fusion CNN			EfficientNetb0		
	COVID-19	Normal	Bacterial	COVID-19	Normal	Bacterial
COVID-19	1653	3	2	1649	4	5
Normal	4	1784	14	5	1771	26
Bacterial	1	9	1790	4	17	1779
	**MobileNetv2**			**Inceptionv3**		
COVID-19	1636	3	19	1632	15	11
Normal	10	1656	136	5	1737	60
Bacterial	25	40	1735	11	38	1751
	**ResNet50**			**ResNet101**		
COVID-19	1460	7	191	1631	7	20
Normal	8	1631	163	7	1740	55
Bacterial	21	29	1750	12	32	1756

CNN: Convolutional neural networks.

**Table 3:** The performance classification by using the Fusion CNN model for each group.

Group	Recall Rate	False Positive Rate	Precision	ROC Area
COVID-19	0.997	0.003	0.997	0.998
Normal	0.994	0.007	0.991	0.994
Bacterial	0.990	0.009	0.993	0.995

**Table 4:** The feature information about the Fusion CNN model and the five individual CNN models used for feature extraction via transferred learning.

Model	Type	Feature Size
Fusion CNN	Transferred Learning + Classifier Base	15 × 4208
EfficientNetb0	Transferred Learning	3 × 4208
MobileNetv2	Transferred Learning	3 × 4208
Inceptionv3	Transferred Learning	3 × 4208
ResNet50	Transferred Learning	3 × 4208
ResNet101	Transferred Learning	3 × 4208

CNN: Convolutional neural networks.

**Table 5:** The results of the presented method were compared to related works.

Author	Year	Method	Dataset	Accuracy	Limitation
Khan et al. [4]	2020	CoroNet (Deep Neural Network)	Chest X-ray images	0.896	Limited dataset
Karar et al. [21]	2021	Cascaded Deep Learning Classifiers	Chest X-ray images	0.999	Complexity of the model
Constantinou et al. [22]	2023	Pre-trained CNNs with Transfer Learning	Chest X-ray images	0.960	Limited generalization capability
Chouat et al. [23]	2022	CT and CXR images with Deep Learning Models	CT and CXR images	0.905	Limited to specific CNN models
Attallah [24]	2023	RADIC (Deep Learning and Quad-Radiomics)	CT and X-ray images	0.994	Complexity and computational cost
Attallah & Samir [25]	2022	Wavelet-based Deep Learning Pipeline	CT images	0.997	Limited to CT slices
Attallah [26]	2022	Texture-based Radiomics Images for COVID-19 Diagnosis	CT images	0.997	Limited to texture-based features
Shankar & Perumal [27]	2021	FM-HCF-DLF Model (Hand-crafted and Deep Learning Features Fusion)	Chest X-ray images	0.941	May not work well on larger datasets
Ragab & Attallah [29]	2020	FUSI-CAD (Fusion of CNNs and Hand-crafted Features)	Chest X-ray images	0.990	Complexity and computational cost
The Presented Method	2023	Fusion CNN Method	Chest X-ray images	0.974	Without Combined CT Images

CT: Computed tomography; CXR: Chest X-ray, CNN: Convolutional neural networks.

## Data Availability

The liver Chest X-ray data set was acquired from at URL: https://www.kaggle.com/datasets/paultimothymooney/chest-xray-pneumonia, accessed on 13 June 2022.
